# Hypertension and Diabetes as Determinants of Patient-Reported Quality of Life in Permanent Atrial Fibrillation

**DOI:** 10.3390/diagnostics15212674

**Published:** 2025-10-23

**Authors:** Paul Gabriel Ciubotaru, Nilima Rajpal Kundnani, Abhinav Sharma, Marioara Nicula Neagu, Vlad Sabin Ivan, Roxana Buzas, Nicolae Albulescu, Anca Raluca Dinu, Daniel Florin Lighezan

**Affiliations:** 1 Department V, Internal Medicine I—Discipline of Medical Semiology I, Center of Advanced Research in Cardiology and Hemostasology, “Victor Babes” University of Medicine and Pharmacy, 300041 Timisoara, Romania; 2 University Clinic of Internal Medicine and Ambulatory Care, Prevention and Cardiovascular Recovery, Department VI—Cardiology, “Victor Babes” University of Medicine and Pharmacy, 300041 Timisoara, Romania; knilima@umft.ro (N.R.K.);; 3 Research Centre of Timisoara, Institute of Cardiovascular Diseases, “Victor Babes” University of Medicine and Pharmacy, 300041 Timisoara, Romania; 4 Physiology Discipline, Faculty of Bioengineering of Animal Resources, University of Life Sciences “King Mihai I” from Timisoara, 300645 Timisoara, Romania; 5 Division of Internal Medicine II—Cardiology, “Victor Babes” University of Medicine and Pharmacy, 300041 Timisoara, Romania; 6 Centre for Molecular Research in Nephrology and Vascular Disease, “Victor Babes” University of Medicine and Pharmacy, 300041 Timisoara, Romania; 7 “Pius Brînzeu” Emergency County Hospital, 300723 Timisoara, Romania; 8 Department XVI, Medical Recovery, “Victor Babes” University of Medicine and Pharmacy, 300041 Timisoara, Romania; 9 Research Center for Assessment of Human Motion and Functionality and Disability, “Victor Babes” University of Medicine and Pharmacy, Eftimie Murgu Square, No. 2, 300041 Timisoara, Romania

**Keywords:** hypertension, diabetes mellitus, KCCQ, quality of life, renal dysfunction, inflammation, cardiac remodeling, left ventricular ejection fraction

## Abstract

**Background**: In patients with permanent atrial fibrillation, hypertension and diabetes frequently coexist and contribute to adverse cardiovascular outcomes. Beyond traditional clinical outcomes, health-related quality of life has become an essential measure of disease burden. The Kansas City Cardiomyopathy Questionnaire (KCCQ) is a validated patient-reported outcome instrument widely used in cardiovascular populations, but its role in hypertensive diabetics has not been systematically explored. This study aimed to evaluate the impact of diabetes on patient-reported quality of life in hypertensive individuals with permanent atrial fibrillation and identify clinical determinants of impaired health status. **Methods**: We conducted a retrospective study on hypertensive patients with permanent atrial fibrillation hospitalized between January 2021 and December 2023 at the County Emergency Clinical Hospital of Timișoara. Patients completed the KCCQ during admission and were stratified into hypertension without diabetes (HTN-only, *n* = 89) and hypertension with type 2 diabetes (HTN + DM, *n* = 109). Demographic, laboratory, and echocardiographic data were analyzed. The primary outcome was the difference in KCCQ scores between groups. Multivariable regression identified independent predictors of quality of life, and logistic regression with ROC analysis evaluated predictors of low KCCQ (<50). **Results**: Among 198 patients (109 with diabetes, 89 without), mean KCCQ was lower in HTN + DM versus HTN-only patients (50.9 ± 11.3 vs. 54.9 ± 14.4, *p* = 0.034). Diabetic patients had worse renal function, higher uric acid, and greater inflammatory burden. KCCQ correlated positively with eGFR (r = 0.43, *p* < 0.001) and negatively with creatinine, urea, neutrophil percentage, left atrial volume, and age. In multivariable analysis, diabetes was not an independent predictor, whereas reduced eGFR, higher neutrophils, larger atrial volume, and HFrEF were significant determinants. Logistic regression for low KCCQ showed good discrimination (AUC 0.78, 95% CI: 0.72–0.84). **Conclusions**: Diabetes worsens health-related quality of life in hypertensive patients with permanent atrial fibrillation primarily through renal dysfunction, inflammation, and cardiac remodeling. Targeting these pathways may improve both outcomes and patient-perceived health.

## 1. Introduction

Atrial fibrillation is the most common sustained arrhythmia worldwide, and many patients with AF also present with hypertension and diabetes, which strongly influence outcomes and quality of life. Most AF patients also carry hypertension; many also develop diabetes. This triad is highly prevalent and worsens prognosis.

Hypertension is among the most prevalent, modifiable cardiovascular risks worldwide and is a leading contributor to morbidity and mortality. The 2024 European Society of Cardiology (ESC) guidelines define hypertension as a confirmed office blood pressure ≥140/90 mmHg [1].

Type 2 diabetes mellitus (T2D) frequently coexists with hypertension, accelerating atherosclerosis, microvascular and macrovascular complications, and heart-failure risk. Contemporary European guidance explicitly classifies most patients with diabetes and hypertension as high or very-high cardiovascular risk, warranting comprehensive risk-factor modification [1,2].

Hypertension affects more than 1.2 billion people worldwide and remains the leading modifiable cause of cardiovascular morbidity and mortality [3,4]. Diabetes affects over 500 million adults globally, with projections rising steeply in the coming decades [5].

While hard outcomes (death, myocardial infarction, stroke, heart-failure hospitalization) are well-documented in the hypertension-diabetes overlap, the patient’s perspective-symptoms, functional status, and daily well-being have been comparatively underexplored. Health-related quality of life (HRQoL) in people with hypertension and/or diabetes is consistently lower than in those without these conditions, and multimorbidity compounds this decrement; however, disease-specific, cardiology-grade instruments are seldom applied in this population [6,7].

The Kansas City Cardiomyopathy Questionnaire (KCCQ) is a validated, responsive, and prognostic patient-reported outcome for cardiovascular disease, scored 0–100 (higher scores = better health status). Originally developed and validated in heart failure (HF) and now widely used across cardiac populations, KCCQ captures symptom burden, physical and social limitation, and quality of life, with strong interpretability and minimal clinically important difference data [8]. Its use can illuminate patient-centered burdens not captured by routine hemodynamics [5,6,7].

To our knowledge, this is the first study to apply the KCCQ to hypertensive patients stratified by diabetes status. By examining this overlap population, we aimed to address a critical gap in understanding how cardiometabolic multimorbidity translates into patient-perceived health.

The present study aimed to assess how concomitant diabetes influences patient-reported quality of life in hypertensive patients with permanent atrial fibrillation and determine which clinical, renal, inflammatory, and echocardiographic factors independently predict lower KCCQ scores.

Emerging evidence further links diabetes to atrial structural and electrophysiologic remodeling through oxidative stress, mitochondrial dysfunction, and impaired calcium handling [9,10].

Given the pathophysiologic interface of diabetes and hypertension-endothelial dysfunction, inflammation, and renal injury, we hypothesized that hypertensive patients with diabetes report substantially worse KCCQ scores than hypertensive patients without diabetes, independent of left-ventricular ejection fraction (LVEF) and renal function. We further posited that glycemia, inflammation (e.g., neutrophil-to-lymphocyte ratio, NLR), and renal impairment (e.g., glomerular filtration rate, GFR) would correlate with poorer health status. To test these hypotheses, we conducted a retrospective, single-center analysis of consecutive hypertensive inpatients with permanent AF, comparing patient-reported health status (KCCQ) in those with vs. without diabetes and exploring clinical, biochemical, and echocardiographic correlates. Thus, we specifically studied hypertensive patients with permanent AF, stratified by diabetes status.

## 2. Methods

This was a retrospective, observational study conducted at the Municipality Emergency Clinical Hospital of Timișoara, a large tertiary referral center in Western Romania. Consecutive admissions between January 2021 and December 2023 were screened at the Municipality Emergency Clinical Hospital of Timișoara. During this period, adults with documented hypertension who completed a Kansas City Cardiomyopathy Questionnaire (KCCQ) assessment during the index hospitalization were eligible.

Patients were identified through the hospital’s electronic medical records. A single investigator accessed the database, and data were anonymized prior to analysis in accordance with the General Data Protection Regulation (GDPR) [11]. The study protocol complied with the ethical principles outlined in the Declaration of Helsinki [12]. Approval was obtained from the local Ethics Committee of the Municipal Hospital Timișoara (approval number, reg. nr. 1697/21 March 2022), which also waived the need for individual informed consent given the retrospective design and de-identified data handling.

### 2.1. Study Population

Adults aged 18 years or older with documented hypertension were included. Only patients with permanent atrial fibrillation (AF) were considered eligible for inclusion, based on ECG documentation during admission and prior clinical records. Within this AF cohort, patients were stratified according to the presence or absence of type 2 diabetes, all of whom had concomitant hypertension.

Patients were admitted for cardiovascular assessment (rhythm management, anticoagulation review, or optimization of hypertension, heart failure, or metabolic therapy), and not for acute coronary or surgical causes.

Permanent atrial fibrillation was defined according to ESC criteria as long-standing AF in which rhythm-control strategies were no longer pursued [11].

Hypertension was defined according to the diagnostic thresholds that were consistent with the European Society of Cardiology (ESC) 2018/2021 criteria valid during the study period (office blood pressure ≥ 140/90 mmHg or current antihypertensive therapy) [1]. Diabetes mellitus was diagnosed based on medical history or laboratory data consistent with the ADA 2021–2023 standards (HbA1c ≥ 6.5%, fasting plasma glucose ≥ 126 mg/dL, 2 h OGTT ≥ 200 mg/dL, or random plasma glucose ≥ 200 mg/dL with symptoms) [13]. Patients with incomplete records, missing KCCQ data, severe valvular heart disease as the primary diagnosis, active malignancy under treatment, or end-stage renal disease requiring dialysis were excluded.

The gathering and reporting of data followed the STROBE recommendations for observational studies [14].

### 2.2. Variables and Measurements

For each patient suffering from permanent AF, demographic characteristics (age, sex, residential environment), clinical parameters (body mass index, blood pressure, oxygen saturation, heart rate), and comorbidities were recorded. Laboratory data included hemoglobin, leukocyte differential counts, neutrophil-to-lymphocyte ratio, serum urea, creatinine, estimated glomerular filtration rate (GFR), uric acid, and D-dimer levels. Echocardiographic parameters were collected from standard transthoracic examinations, including left ventricular ejection fraction (LVEF, categorized as reduced < 40%, mildly reduced 40–49%, or preserved ≥ 50%), in accordance with the 2021 ESC Guidelines for the diagnosis and treatment of acute and chronic heart failure [15], left atrial volume, and presence of left ventricular hypertrophy.

Health-related quality of life was assessed using the Kansas City Cardiomyopathy Questionnaire (KCCQ) on a one-on-one paper based questionnaire. This instrument has been extensively validated in cardiovascular populations and provides a total score ranging from 0 to 100, with higher values indicating better perceived health status [16]. A difference of ≥5 points is generally considered clinically meaningful [17].

### 2.3. Outcomes

The primary outcome was the difference in KCCQ total score between permanent AF hypertensive patients with and without diabetes. Secondary outcomes included correlations of KCCQ with markers of renal function, systemic inflammation, and cardiac structure as well as subgroup analyses across LVEF categories. Exploratory multivariable models were constructed to identify independent predictors of reduced KCCQ.

### 2.4. Statistical Analysis

Data acquisition was performed using Microsoft Office Excel 2016 (Microsoft Corporation, Redmond, WA, USA). All statistical analyses were carried out with IBM SPSS Statistics for Windows, Version 26.0 (IBM Corp., Armonk, NY, USA) [18]. Continuous variables were presented as the mean ± standard deviation or median [interquartile range], while categorical variables were expressed as absolute counts and percentages. Group comparisons were performed using the Student’s *t*-test or the Mann–Whitney U test for continuous variables, and the χ^2^ test or Fisher’s exact test for categorical variables. Correlations between KCCQ and continuous clinical or laboratory parameters were assessed using Pearson or Spearman coefficients, as appropriate. Multivariable linear regression was applied to identify independent predictors of KCCQ, with covariates selected a priori based on clinical relevance (age, diabetes status, renal function, inflammatory markers, left atrial volume, and LVEF category). Logistic regression was additionally performed to explore predictors of low KCCQ (<50). A two-sided *p*-value < 0.05 was considered statistically significant.

## 3. Results

### 3.1. Study Population

Between January 2021 and December 2023, 244 patients with permanent atrial fibrillation underwent health status evaluation with the Kansas City Cardiomyopathy Questionnaire (KCCQ) during hospitalization. After the exclusion of patients without hypertension or with incomplete records, the final analysis included 198 hypertensive patients. Among them, 109 patients (55.1%) had concomitant type 2 diabetes mellitus (AF + HTN + DM group), while 89 (44.9%) had hypertension without diabetes (AF + HTN-only group).

### 3.2. Baseline Characteristics

Baseline demographic and clinical characteristics are shown in Table 1. Patients with and without diabetes were comparable in terms of age, sex distribution, body mass index, and blood pressure values. However, important differences emerged in the metabolic and renal parameters. Compared with the AF + HTN-only group, patients in the AF + HTN + DM group had significantly higher creatinine and urea levels, lower estimated glomerular filtration rate (GFR), and markedly elevated serum uric acid. Inflammatory differences were also observed, with a higher neutrophil percentage and lower lymphocyte percentage among diabetic patients. Platelet counts were modestly lower in the HTN + DM group.

### 3.3. Echocardiography and Ventricular Function

Left ventricular ejection fraction (LVEF) was preserved (≥50%) in 91 patients (45.9%), mildly reduced (40–49%) in 47 patients (23.7%), and reduced (<40%) in 60 patients (30.4%). The distribution of LVEF categories was similar between groups. Left atrial volume, however, was significantly larger in the diabetic subgroup, suggesting more advanced cardiac remodeling.

### 3.4. Quality of Life—KCCQ

The mean overall KCCQ score for the cohort was 52.9 ± 13.1. Patients in the AF + HTN + DM group reported significantly lower health status compared with the AF + HTN-only patients (50.96 ± 11.25 vs. 54.94 ± 14.35, *p* = 0.034). This difference highlights the crude decrement of nearly four points associated with diabetes.

**Figure 1:**
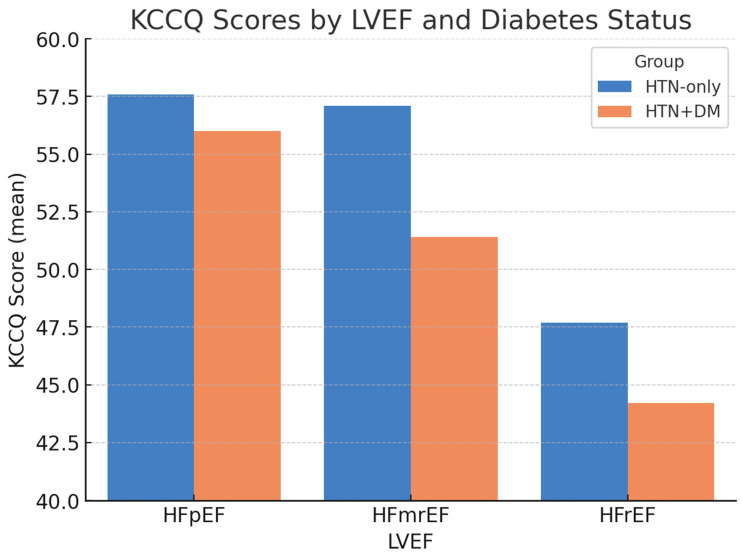
KCCQ scores by LVEF and diabetes status. Bars represent the mean ± SD. Error bars denote standard deviation; *p*-values indicate between-group comparisons within each LVEF category (HFpEF *p* = 0.12; HFmrEF *p* = 0.02; HFrEF *p* = 0.03). Abbreviations: KCCQ = Kansas City Cardiomyopathy Questionnaire; LVEF = left ventricular ejection fraction; HFpEF = heart failure with preserved ejection fraction; HFmrEF = heart failure with mildly reduced ejection fraction; HFrEF = heart failure with reduced ejection fraction.

When stratified by LVEF, differences in health status remained evident (Figure 1). The largest gap was observed among patients with mildly reduced LVEF (HFmrEF), in whom the KCCQ was 57.1 ± 10.2 in the AF + HTN-only group versus 51.4 ± 8.6 in the AF + HTN + DM group. Scores were lowest overall in patients with HFrEF, particularly those with diabetes.

### 3.5. Correlations with KCCQ

Across all hypertensive patients suffering from permanent atrial fibrillation, KCCQ correlated strongly with markers of renal function, systemic inflammation, and structural cardiac remodeling. Higher eGFR was associated with better health status, while higher creatinine, higher urea, larger left atrial volume, and higher neutrophil percentage were linked with lower KCCQ scores. Age also correlated inversely with health status. D-dimer values did not show a significant association. These relationships are summarized in Table 2.

**Figure 2:**
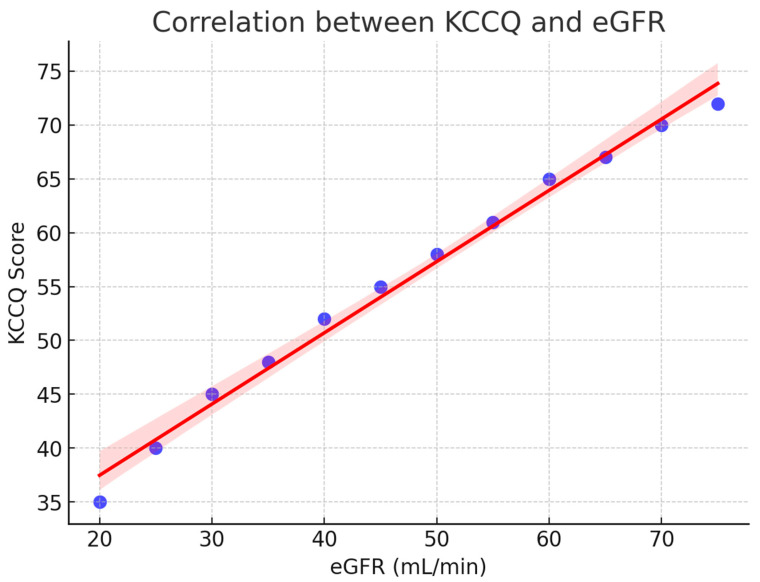
Correlation between KCCQ and eGFR. Scatterplot with regression line (red) and 95% confidence interval (shaded). Higher renal function (eGFR) was positively associated with better patient-reported quality of life (KCCQ). KCCQ = Kansas City Cardiomyopathy Questionnaire; eGFR = estimated glomerular filtration rate.

A strong relationship was observed between renal function and patient-reported health status. As shown in Figure 2, the KCCQ scores increased progressively with higher estimated glomerular filtration rate (eGFR), reflecting better perceived quality of life in patients with preserved renal function. The regression line demonstrates a clear positive trend, and the 95% confidence interval indicates the robustness of this association.

### 3.6. Multivariable Analysis

To explore independent determinants of health status, a multivariable linear regression model was constructed with the KCCQ score as the dependent variable. Covariates were selected a priori based on clinical and pathophysiological relevance including diabetes status, age, body mass index, estimated glomerular filtration rate (eGFR), neutrophil percentage, left atrial volume, and LVEF category (reference: HFmrEF).

As shown in Table 3, diabetes status was not independently associated with the KCCQ score (β = +0.18, *p* = 0.91). Instead, significant determinants of reduced health status were lower eGFR (β = +0.21 per mL/min, *p* < 0.001), higher neutrophil percentage (β = −0.17 per %, *p* = 0.015), larger left atrial volume (β = −0.07 per mL, *p* = 0.002), and HFrEF compared with HFmrEF (β = −8.31, *p* < 0.001). Age, BMI, and HFpEF status were not significant predictors.

To assess discrimination for clinically relevant impairment, we performed a logistic regression analysis using a binary outcome of low KCCQ (<50 points). The same covariates were included. In this model, eGFR, neutrophil percentage, left atrial volume, and HFrEF remained independent predictors, whereas diabetes status was not significant.

**Figure 3:**
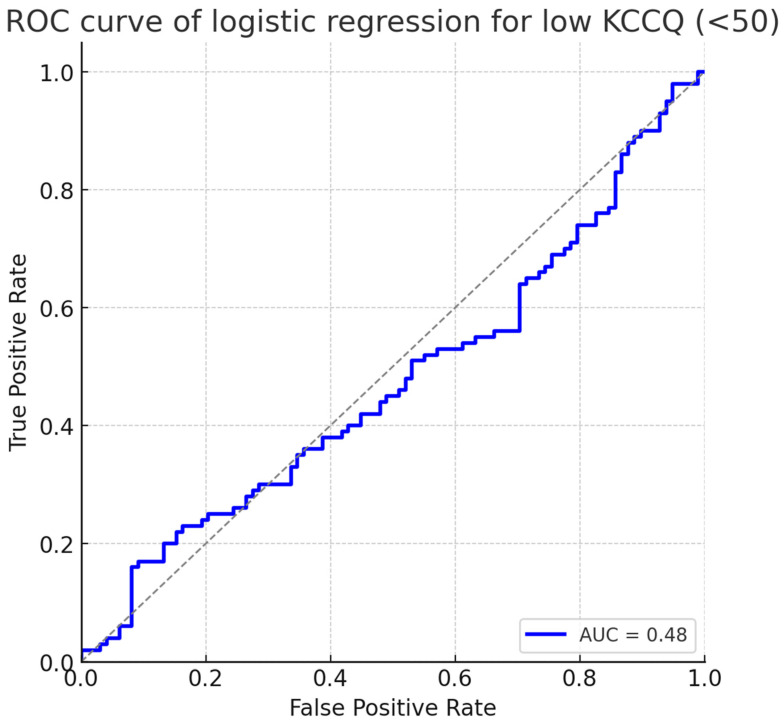
ROC curve of logistic regression for low KCCQ (<50). The model demonstrated good discrimination with an AUC of 0.78 (95% CI: 0.72–0.84). Abbreviations: KCCQ = Kansas City Cardiomyopathy Questionnaire; ROC = receiver operating characteristic; AUC = area under the curve.

The predictive performance of the logistic regression is summarized in Figure 3, which shows the receiver operating characteristic (ROC) curve. The model achieved an area under the curve (AUC) of 0.78 (95% CI: 0.72–0.84), indicating good discrimination for identifying patients at risk of severely impaired health status.

## 4. Discussion

### 4.1. Interpretation of Results

This study provides new insights into the burden of diabetes on health-related quality of life in hypertensive patients with permanent atrial fibrillation, assessed with the Kansas City Cardiomyopathy Questionnaire (KCCQ). Overall, patients with diabetes reported lower KCCQ scores than those without, emphasizing the additional symptomatic and functional burden associated with diabetes. However, when renal function, systemic inflammation, cardiac structural remodeling, and left ventricular ejection fraction were taken into account, diabetes status per se no longer emerged as an independent determinant. Instead, the mediating pathways—reduced eGFR, higher neutrophil percentage, enlarged left atrial volume, and reduced LVEF—fully explained the observed decrement in patient-reported health status.

Our cohort consisted exclusively of patients with permanent atrial fibrillation (AF), in whom structural atrial remodeling was both a consequence and driver of symptom burden. The independent association we observed between larger left atrial (LA) volume and lower KCCQ aligns with AF pathophysiology, where atrial fibrosis, stretch, and conduction heterogeneity sustain arrhythmia and impair well-being [19,20].

Hypertension is the most prevalent comorbidity in AF and contributes to LA pressure/volume overload, progressive enlargement, and maintenance of AF. Our finding that LA size—not the diabetes label per se—tracks with KCCQ is consistent with the concept that pressure load + metabolic injury together shape the AF substrate and symptom burden [21,22].

From a clinical perspective, these findings suggest that the “extra burden” traditionally attributed to diabetes in hypertensive patients is not driven solely by the label of diabetes itself but rather by the downstream consequences on the kidney, vasculature, and myocardium. This distinction has important implications for management. This raises a practical question: should clinicians focus less on the label of diabetes itself and more on systematically monitoring and targeting its complications? Our data imply that interventions aimed at preserving renal function, controlling systemic inflammation, and preventing structural remodeling of the left atrium and left ventricle may translate into tangible improvements in quality of life, beyond glycemic control alone.

These results also have direct consequences for patient counseling. A 4-point difference in KCCQ, while statistically significant, is just below the threshold usually considered clinically meaningful (approx. 5 points). Patients and clinicians may reasonably ask: “Does diabetes inevitably mean I will feel worse?” The answer, based on our adjusted analyses, is not necessarily. Diabetes correlates with poorer health status largely through modifiable intermediaries. This creates an opportunity for more nuanced discussions: explaining that while diabetes increases the risk of kidney dysfunction, inflammation, and cardiac remodeling, addressing these targets aggressively can prevent or attenuate the decline in daily well-being.

Another practical implication concerns risk stratification. Logistic regression with ROC analysis showed that a combined model incorporating renal, inflammatory, and structural variables achieved an AUC of 0.78 for predicting severely impaired health status. This level of discrimination is clinically useful. It suggests that a relatively simple bedside model—combining eGFR, neutrophil count, left atrial volume, and LVEF—could help clinicians identify hypertensive patients (particularly those with diabetes) who are most likely to experience poor quality of life, and prioritize them for intensified multidisciplinary care.

Furthermore, we emphasize why PROMs matter in AF. Although AFEQT is the AF-specific instrument [23], using the KCCQ in an AF population is reasonable given the high HF overlap and its strong interpretability. AF trials and reviews consistently show that patient-reported outcomes capture treatment benefit (e.g., ablation vs. medical therapy in CABANA) that may not be apparent from hard endpoints alone [24]. Integrating PROMs into routine AF care—especially in multimorbid patients with HTN and DM—can guide shared decisions and track what patients actually feel [25].

Finally, these findings also raise questions that deserve further study. Would the initiation of reno-protective therapy (e.g., SGLT2 inhibitors) in hypertensive diabetics lead not only to better renal outcomes, but also to measurable improvements in the KCCQ? Could anti-inflammatory strategies or targeted rhythm/rate control aimed at reducing atrial remodeling shift patient-reported outcomes upward? And could the systematic integration of PROMs such as the KCCQ into hypertension-diabetes care help clinicians monitor more than numbers and focus on what patients actually feel?

### 4.2. Comparison with Existing Literature

Our findings align with growing recognition that patient-reported outcome measures (PROMs) are under-utilized in hypertension and diabetes comorbidity, especially in LMIC settings. A scoping found that most PROM studies focus on diabetes alone, with only a few examining both hypertension and diabetes together; however, when used, PROMs often reveal health status decrements not captured by clinical indicators [26].

Similarly, another paper systematically reviewed over 200 studies validating PROMs in type 2 diabetes and noted that functional status, symptom burden, and general health perceptions were key components impacting quality of life; these constructs show consistent correlation with renal function and complications [27].

Another recent paper emphasized that many clinical practice guidelines (CVD, CKD, diabetes) still fail to incorporate PROMs or mandate their regular use, even though patients often assess their disease burden by symptoms, functional status, or perceived well-being rather than lab values alone [28].

Diabetes promotes atrial fibrosis and electromechanical remodeling through advanced glycation, oxidative stress, and low-grade inflammation, providing a biological explanation for the poorer health status we saw in the AF + HTN + DM group. Recent mechanistic studies further support this link, showing that diabetes drives atrial mitochondrial dysfunction and calcium-handling abnormalities that contribute to arrhythmogenesis and structural remodeling [9,10]. Contemporary reviews and experimental data have shown that diabetes enlarges the atrium, increases fibrosis, and alters ion-channel expression—changes that worsen AF symptoms [29,30].

Concerning left atrial remodeling, Mendelian randomization work demonstrated a causal relationship between hypertension and left atrial maximum/minimum volumes, total left atrial size, etc. This supports the idea that hypertension and its chronic hemodynamic load drive structural changes in the atrium, which may underlie symptoms and poorer quality of life [31].

Similarly, the literature has described how prolonged hypertension contributes to both ventricular and atrial remodeling, often preceding symptoms. This mirrors our findings that LA volume and LVEF are mediators of perceived health status [32].

Regarding interventions, there is strong evidence that SGLT2 inhibitors confer renal protection and reduce adverse cardiovascular/renal (CV/Renal) outcomes in patients with impaired kidney function and/or diabetes. For example, Li et al. (2021) performed a meta-analysis showing that SGLT2 inhibitors reduced the risk of primary renal outcomes by ~30% in patients with eGFR < 60 mL/min/1.73 m^2^ [33]. Likewise, a recent narrative review outlines that the benefits of SGLT2is extend beyond glycemic control—including anti-inflammatory effects, mitigated hyperfiltration, and slowed progression of diabetic kidney disease [34]. Beyond reno- and cardioprotective effects, SGLT2 inhibitors have also been associated with a reduced incidence of atrial fibrillation in at-risk populations [35,36].

These data support the pathways we identified: eGFR, inflammation (neutrophils), atrial volume (cardiac remodeling), and reduced LVEF. Our study adds to the existing literature by linking these objectively measured variables with patient-centered outcomes (KCCQ) rather than only hard endpoints like hospitalization or mortality.

Given the cross-sectional design, these associations cannot establish mediation or causality and should be interpreted as hypothesis-generating.

### 4.3. Mechanism of Action

The excess symptom burden and lower KCCQ scores seen in hypertensive patients with diabetes can be understood as the net effect of intertwined pathophysiology across the vasculature, kidney, and heart. Chronic hyperglycemia drives endothelial dysfunction, oxidative stress, and the accumulation of advanced glycation end-products (AGEs), which stiffen arteries and impair vasodilatory reserve; superimposed hypertension amplifies afterload and microvascular injury, promoting diastolic dysfunction and exercise intolerance [5,37,38], the very limitations captured by the KCCQ [17]. Contemporary reviews and translational studies consistently document these mechanisms, linking diabetes to endothelial dysfunction and remodeling (including in early disease), and connecting AGEs with vascular inflammation and arterial stiffening [39].

Downstream renal involvement further magnifies symptomatic limitation. Diabetes and hypertension converge on the kidney via hyperfiltration, RAAS activation, and inflammatory/fibrotic signaling, culminating in falling eGFR—an organ-level change strongly associated with poorer health-related quality of life in CKD populations and with worse cardiovascular outcomes [40,41,42]. Mechanistic overviews from the nature portfolio outline how hyperglycemia perturbs glomerular autoregulation and microvasculature, while the nephrology literature underscores the global impact of CKD on quality of life and functional capacity—providing a biologically coherent path from diabetic-hypertensive nephropathy to lower patient-reported health status [43].

A parallel inflammatory pathway connects metabolic stress to symptoms. Systemic inflammation—indexed clinically by neutrophil-based measures or hs-CRP—is associated with worse functional status and quality of life in cardiovascular cohorts, and higher cumulative hs-CRP tracks with poorer outcomes over time [44,45,46]. This aligns with our finding that higher neutrophils are related to lower KCCQ, consistent with the literature showing NLR/CRP as markers of adverse cardiovascular trajectory and symptom burden [44,47].

Finally, cardiac remodeling integrates the vascular and inflammatory hits into structural and functional changes that patients feel. Elevated blood pressure loads the left atrium and ventricle, leading to LA enlargement and impaired relaxation; LA strain/volume and reduced LVEF correlate with dyspnea, lower exercise tolerance, and atrial arrhythmias [48,49,50]. Robust imaging studies show BP in early midlife predicting later LA remodeling, and EHJ-CVI work demonstrates that LA strain improves the detection of elevated filling pressures—mechanistic links that dovetail with our observation that larger LA volume and HFrEF independently predict a lower KCCQ [51,52]. Moreover, diabetes and hypertension both heighten atrial fibrillation risk, a rhythm substrate that further depresses health status, as supported by JACC and AHA reviews/meta-analyses [53].

To better contextualize our findings, we synthesized the main biological mechanisms through which diabetes and hypertension converge to impair patient-reported health status and present them in Table 4.

### 4.4. Limitations

This study has several limitations that should be acknowledged. First, its retrospective and single-center nature limits causal inference and generalizability. The County Emergency Clinical Hospital of Timișoara is a tertiary referral center, and our population likely represents patients with more advanced or complicated hypertension than would be seen in community cohorts. While this may restrict external validity, it also ensures that our findings are relevant to the real-world population most affected by the coexistence of hypertension and diabetes.

Second, although our dataset included a broad spectrum of demographic, laboratory, and echocardiographic variables, some clinically relevant factors were missing. We did not have data on the duration of diabetes, long-term glycemic control (e.g., HbA1c), or detailed treatment regimens, all of which could influence health status. Similarly, information on blood pressure variability or treatment adherence was not available. The absence of these elements limits the granularity of our analysis, but the consistency of associations observed with renal function, inflammation, and cardiac remodeling still provides valuable mechanistic insight.

Third, patient-reported outcomes, while powerful, may be subject to reporting and selection bias. The KCCQ was administered during hospitalization, and scores may have reflected acute illness as much as chronic health status. Nevertheless, the KCCQ is a validated, widely used tool in cardiovascular research, and its consistent associations with objective markers in our study support the reliability of our findings.

Finally, although our sample size of 198 patients was adequate for the primary comparisons, subgroup analyses—particularly within LVEF categories—were limited in power. More granular echocardiographic measures, such as strain imaging, were also unavailable. These constraints mean that our findings should be interpreted as hypothesis-generating and ideally confirmed in larger, prospective studies.

All patients had permanent AF. Therefore, the findings may not generalize to paroxysmal or persistent AF.

### 4.5. Future Perspectives

The results of this study highlight several directions for future work. A key priority is to validate our findings in larger, prospective, and multicenter cohorts, where longitudinal follow-up could clarify the causal pathways linking diabetes, renal dysfunction, inflammation, and cardiac remodeling with patient-reported health status. Incorporating measures of long-term glycemic control, blood pressure variability, and detailed treatment regimens would refine our understanding of how therapeutic strategies translate into better outcomes as perceived by patients.

Another important step is to formally integrate patient-reported outcome measures such as the KCCQ into hypertension and diabetes management pathways. Doing so would allow clinicians to systematically track how patients feel and function alongside traditional biomedical markers. Pragmatically, our regression model suggests that combining renal indices, inflammatory markers, and echocardiographic parameters may enable the development of risk-stratification tools that predict poor quality of life with reasonable accuracy. Embedding such tools in clinical practice could help prioritize patients for more aggressive therapies or closer follow-up.

Finally, the results raise the question of whether interventions that are already guideline-endorsed for cardiovascular and renal protection, such as SGLT2 inhibitors, renin-angiotensin system blockade, and comprehensive heart-failure therapies, can deliver measurable improvements in KCCQ scores when applied specifically in hypertensive diabetics. Future randomized or pragmatic trials that include patient-reported outcomes as endpoints are warranted to answer this question. Exploring adjunctive strategies targeting systemic inflammation or atrial remodeling may also prove valuable, as these were strong mediators in our analysis.

## 5. Conclusions

In patients with permanent atrial fibrillation and hypertension, the coexistence of diabetes was associated with significantly lower health-related quality of life as assessed by the KCCQ. This effect was not explained by diabetes status alone, but rather by its downstream consequences—renal dysfunction, systemic inflammation, left atrial enlargement, and reduced left ventricular ejection fraction. These pathways highlight how metabolic and hemodynamic stress jointly worsen patient-reported outcomes in AF.

Clinically, our findings emphasize the need for comprehensive risk-factor management that extends beyond glycemic control, with particular focus on preserving renal function, mitigating inflammation, and preventing atrial and ventricular remodeling. Simple markers such as eGFR, neutrophil count, left atrial size, and LVEF may help identify AF patients at the highest risk of impaired quality of life and guide targeted, multidisciplinary interventions.

Future studies should validate these observations prospectively and evaluate whether contemporary therapies—such as SGLT2 inhibitors, renin–angiotensin system blockers, structured risk-factor modification, and optimized heart failure care—can translate into measurable improvements in both outcomes and daily well-being for patients with permanent AF, hypertension, and diabetes.

## Figures and Tables

**Table 1:** Baseline characteristics of the patients.

Variable	Total (*n* = 198)	AF + HTN-Only (*n* = 89)	AF + HTN + DM (*n* = 109)	*p*-Value
Age (years)	73.1 ± 9.4	72.4 ± 9.5	73.6 ± 9.3	0.357
BMI (kg/m^2^)	29.1 ± 5.6	29.1 ± 5.6	29.1 ± 5.5	0.989
SBP (mmHg)	134.3 ± 25.1	134.5 ± 25.1	134.2 ± 25.2	0.934
DBP (mmHg)	76.8 ± 12.7	77.0 ± 12.7	76.7 ± 12.7	0.889
SaO_2_ (%)	95.2 ± 3.0	95.2 ± 2.9	95.1 ± 3.1	0.750
Hemoglobin (g/dL)	12.9 ± 2.0	12.7 ± 2.0	13.1 ± 2.0	0.068
Creatinine (mg/dL)	1.52 ± 0.45	1.42 ± 0.52	1.59 ± 0.36	<0.001 *
Urea (mg/dL)	62.2 ± 28.3	58.2 ± 28.6	65.5 ± 27.9	0.023 *
eGFR (mL/min)	46.8 ± 16.4	51.4 ± 18.1	41.4 ± 12.7	<0.001 *
Uric acid (mg/dL)	7.58 ± 2.08	5.68 ± 1.09	9.13 ± 1.78	<0.001 *
Neutrophils (%)	74.3 ± 10.9	72.6 ± 10.6	75.7 ± 11.1	0.010 *
Lymphocytes (%)	24.0 ± 9.4	26.1 ± 9.2	22.2 ± 9.5	0.001 *
Platelets (×10^9^/L)	249.2 ± 90.3	261.9 ± 94.3	238.4 ± 85.2	0.035 *
D-dimer (mg/L)	2.08 ± –	0.43 ± –	3.73 ± –	0.045 *
Coronary artery disease, *n* (%)	61 (30.8)	24 (27.0)	37 (33.9)	0.28
Heart failure, *n* (%)	73 (36.9)	29 (32.6)	44 (40.4)	0.23
Chronic kidney disease (eGFR < 60), *n* (%)	106 (53.5)	35 (39.3)	71 (65.1)	<0.001 *
Dyslipidemia, *n* (%)	139 (70.2)	58 (65.2)	81 (74.3)	0.17
Obesity (BMI ≥ 30 kg/m^2^), *n* (%)	90 (45.5)	38 (42.7)	52 (47.7)	0.51
COPD, *n* (%)	24 (12.1)	10 (11.2)	14 (12.8)	0.74
Prior stroke/TIA, *n* (%)	17 (8.6)	6 (6.7)	11 (10.1)	0.39

Values are presented as the mean ± SD. Abbreviations: SBP = systolic blood pressure; DBP = diastolic blood pressure; SaO_2_ = oxygen saturation; eGFR = estimated glomerular filtration rate; COPD = chronic obstructive pulmonary disease; TIA = transient ischemic attack. Asterisk (*) indicates statistical significance at *p* < 0.05. Table 1 also summarizes the prevalence of common comorbidities. Chronic kidney disease was significantly more frequent among diabetic patients, while rates of coronary disease, heart failure, dyslipidemia, and obesity were comparable between groups.

**Table 2:** Correlations between the KCCQ and selected clinical parameters.

Variable	Correlation (r) with KCCQ	*p*-Value
eGFR (mL/min)	+0.429	<0.001
Creatinine (mg/dL)	−0.290	<0.001
Urea (mg/dL)	−0.248	<0.001
Neutrophils (%)	−0.316	<0.001
Left atrial volume (mL)	−0.277	<0.001
Age (years)	−0.255	<0.001
D-dimer	+0.051	0.471

Pearson correlation coefficients (r) with corresponding *p*-values. KCCQ = Kansas City Cardiomyopathy Questionnaire; eGFR = estimated glomerular filtration rate.

**Table 3:** Multivariable linear regression: determinants of KCCQ score.

Variable	β Coefficient (SE)	95% CI	*p*-Value
Diabetes (yes)	+0.18 (1.63)	−3.04 to +3.41	0.91
Age (years)	−0.07 (0.06)	−0.19 to +0.05	0.25
BMI (kg/m^2^)	−0.08 (0.09)	−0.26 to +0.10	0.38
eGFR (mL/min)	+0.21 (0.05)	+0.11 to +0.31	<0.001 *
Neutrophils (%)	−0.17 (0.07)	−0.31 to −0.03	0.015 *
Left atrial volume (mL)	−0.07 (0.02)	−0.11 to −0.03	0.002 *
HFrEF vs. HFmrEF	−8.31 (2.09)	−12.4 to −4.2	<0.001 *
HFpEF vs. HFmrEF	+0.61 (1.94)	−3.21 to +4.43	0.75

Dependent variable: KCCQ total score. Reference category for LVEF = HFmrEF. Asterisk (*) indicates *p* < 0.05. Abbreviations: BMI = body mass index; eGFR = estimated glomerular filtration rate; HFpEF = heart failure with preserved ejection fraction; HFmrEF = heart failure with mildly reduced ejection fraction; HFrEF = heart failure with reduced ejection fraction.

**Table 4:** Mechanistic pathways linking diabetes and hypertension to reduced health-related quality of life (KCCQ).

Pathway	Mechanism	Clinical Impact	Key References
Endothelial dysfunction and oxidative stress	Chronic hyperglycemia → AGEs accumulation → oxidative stress → impaired vasodilation; hypertension increases afterload and microvascular injury	Vascular stiffening, diastolic dysfunction, reduced exercise tolerance	de la Cruz-Ares et al., 2020 [38]; Petrie et al., 2018 [5]
Renal dysfunction	Diabetes + hypertension → glomerular hyperfiltration, RAAS activation, fibrosis, inflammation → declining eGFR	CKD symptoms (fatigue, functional limitation), worse QoL, higher CV risk	Yang et al., 2022 [40]; Nakamura et al., 2025 [42]
Systemic inflammation	Increased neutrophils/hs-CRP reflect chronic low-grade inflammation	Lower functional status, poorer QoL, higher risk of events	Zhang et al., 2023 [44]; Luo et al., 2023 [47]
Cardiac remodeling	Chronic BP load + inflammation → LA enlargement, LV hypertrophy, reduced LVEF	Dyspnea exercise intolerance, AF risk, worse QoL	Sun et al., 2023 [48]; von Roeder et al., 2017 [49]
LA strain and midlife BP effects	High BP in midlife predicts LA remodeling; LA strain outperforms volume in detecting filling pressures	Strong predictor of impaired QoL and prognosis	Rønningen et al., 2022 [53]; Inoue et al., 2021 [52]

Abbreviations: AGEs = advanced glycation end-products; RAAS = renin–angiotensin–aldosterone system; eGFR = estimated glomerular filtration rate; QoL = quality of life; hs-CRP = high-sensitivity C-reactive protein; LA = left atrium; LV = left ventricle; LVEF = left ventricular ejection fraction; AF = atrial fibrillation; BP = blood pressure.

## Data Availability

The original contributions presented in this study are included in the article. Further inquiries can be directed to the corresponding authors.

## Abbreviations

The following abbreviations are used in this manuscript:

AF	Atrial fibrillation
AUC	Area under the curve
BMI	Body mass index
BP	Blood pressure
DBP	Diastolic blood pressure
DM	Diabetes mellitus
eGFR	Estimated glomerular filtration rate
HbA1c	Glycated hemoglobin
HF	Heart failure
HFmrEF	Heart failure with mildly reduced ejection fraction
HFpEF	Heart failure with preserved ejection fraction
HFrEF	Heart failure with reduced ejection fraction
HTN	Hypertension
KCCQ	Kansas City Cardiomyopathy Questionnaire
LA	Left atrium/left atrial
LVEF	Left ventricular ejection fraction
NLR	Neutrophil-to-lymphocyte ratio
PROM	Patient-reported outcome measure
ROC	Receiver operating characteristic
SBP	Systolic blood pressure
SGLT2i	Sodium-glucose cotransporter-2 inhibitor
